# Evidence that Plasmid-Borne Botulinum Neurotoxin Type B Genes Are Widespread among *Clostridium botulinum* Serotype B Strains

**DOI:** 10.1371/journal.pone.0004829

**Published:** 2009-03-16

**Authors:** Giovanna Franciosa, Antonella Maugliani, Concetta Scalfaro, Paolo Aureli

**Affiliations:** Department of Food Safety and Veterinary Public Health, Unit of Microorganisms and Food Technologies, Istituto Superiore di Sanità, Rome, Italy; Max Planck Institute for Infection Biology, Germany

## Abstract

**Background:**

Plasmids that encode certain subtypes of the botulinum neurotoxin type B have recently been detected in some *Clostridium botulinum* strains. The objective of the present study was to investigate the frequency with which plasmid carriage of the botulinum neurotoxin type B gene (*bont*/B) occurs in strains of *C. botulinum* type B, Ab, and A(B), and whether plasmid carriage is *bont*/B subtype-related.

**Methodology/Principal Findings:**

PCR-Restriction fragment length polymorphism was employed to identify subtypes of the *bont*/B gene. Pulsed-field gel electrophoresis and Southern blot hybridization with specific probes were performed to analyze the genomic location of the *bont*/B subtype genes. All five known *bont*/B subtype genes were detected among the strains; the most frequently detected subtype genes were *bont*/B1 and /B2. Surprisingly, the *bont*/B subtype gene was shown to be plasmid-borne in >50% of the total strains. The same *bont*/B subtype gene was associated with the chromosome in some strains, whereas it was associated with a plasmid in others. All five known *bont*/B subtype genes were in some cases found to reside on plasmids, though with varying frequency (e.g., most of the *bont*/B1 subtype genes were located on plasmids, whereas all but one of the *bont*/B2 subtypes were chromosomally-located). Three bivalent isolates carried both *bont*/A and /B genes on the same plasmid. The plasmids carrying the *bont* gene were five different sizes, ranging from ∼55 kb to ∼245 kb.

**Conclusions/Significance:**

The unexpected finding of the widespread distribution of plasmids harboring the *bont*/B gene among *C. botulinum* serotype B strains provides a chance to examine their contribution to the dissemination of the *bont* genes among heterogeneous clostridia, with potential implications on issues related to pathogenesis and food safety.

## Introduction

Botulinum neurotoxins (BoNTs) are zinc-dependent metalloproteases that inhibit the release of the neurotransmitter acetylcholine from peripheral cholinergic synapses. Through this mechanism of action, they can cause the flaccid paralysis of botulism in humans exposed through foodborne intoxication or intestinal and wound toxemias, thus representing a serious pathogenic and biodefense threat. However, when properly administered they can also provide therapeutic and cosmetic benefits in conditions characterized by excessive cholinergic activity [1].

Seven structurally similar but serologically distinct BoNT variants (A to G) have been identified: of these, BoNT/A and /B are the most frequent causes of human botulism worldwide, and both are used as therapy against a wide variety of involuntary muscle disorders [1], [2]. Recently, comparison of the available BoNT gene and protein sequences has revealed that a certain variability exists within individual BoNT serotypes [3], [4]. In particular, for six of the seven BoNT serotypes (all but serotype G), subtypes that diverge at the amino-acid level by at least 2.6% have been defined: for example, BoNT/A has four distinct subtypes which differ from each other at the amino-acid level by up to 16% (BoNT/A1, /A2, /A3, and /A4), and BoNT/B has five subtypes, differing by up to 6% (BoNT/B1, /B2, /B3, non-proteolytic B, and bivalent B) [4].

All BoNT serotypes and subtypes are encoded by specific *bont* genes that may reside in the genome of six heterogeneous clostridia groups, comprising at least four distinct species (i.e., *Clostridium botulinum*, *C. argentinense*, *C. barati*, and *C. butyricum*) [2]. The *bont*/C and /D genes are located on distinct bacteriophages [5], [6], and the *bont*/G gene is located on a plasmid [7], [8], whereas the *bont*/A, /B, /E and /F genes have long been assumed to be chromosomally located. The genome sequencing of the BoNT/A1-producing *C. botulinum* strain Hall (ATCC 3502) confirmed the chromosomal location of the *bont*/A1 subtype gene [9]. However, unexpectedly, the *bont*/A3 and *bont*/A4 subtype genes of the only two BoNT/A3- and /A4-producing strains identified to date (i.e., Loch Maree and 657) were recently shown to reside on large molecular weight plasmids (∼267 kb and 270 kb, respectively) [10]. Strain 657 forms greater amounts of bivalent BoNT/B than of BoNT/A4 and is thus regarded as *C. botulinum* type Ba [11]: its bivalent *bont*/B subtype gene was shown to reside on the same 270 kb plasmid that carries the *bont*/A4 subtype gene [10]. Nucleotide sequencing confirmed these results and also showed that the *bont*/B1 subtype gene of the *C. botulinum* strain Okra was located on a ∼149 kb plasmid; the plasmids described in strains Loch Maree, 657, and Okra have been designated as “pCLK”, “pCLJ”, and “pCLD”, respectively (GenBank Accession numbers CP000940 and CP000963) [12]. A ∼48 kb plasmid (pCLL) from strain Eklund 17B (ATCC 25765) containing the non-proteolytic *bont*/B subtype gene has been sequenced by the Los Alamos National Laboratory of the United States (GenBank Accession number CP001057), but has not been published to date.

These findings challenge the previous assumption of a chromosomal location of the *bont*/A and /B genes and raise questions as to whether plasmid-borne *bont* genes are rare or widespread among botulinum neurotoxigenic clostridia.

BoNT/B is the most frequent cause of human botulism in Europe (including Italy) and the second leading cause of botulism in North America [13], [14]. The purpose of the present study was to evaluate the genomic location of subtypes of the *bont*/B gene in a panel of *C. botulinum* strains of different origins. We also investigated the association between certain subtypes of the *bont*/B gene and plasmid carriage. The results are the first evidence that all five known *bont*/B subtype genes can reside on plasmids that vary considerably in size, and they clearly indicate that plasmid-borne *bont*/B genes are more widespread among *C. botulinum* serotype B isolates than previously known.

## Results

### PCR-Restriction Fragment Length Polymorphism (RFLP) subtyping of the *bont*/B gene

Based on the alignment of sequences of the *bont*/B gene (3876 bp) present in the GenBank database, the region encompassing nucleotides 920 to 3727 was selected for PCR amplification because it has shown a certain degree of nucleotide polymorphism. This region mainly encodes the transmembrane and receptor binding domains of the neurotoxin, where most of the amino-acid differences among the BoNT/B subtypes have been reported [3]. Analysis of the 2807 bp region from the five distinct *bont*/B subtypes using the restriction mapper program Webcutter 2.0 (http://rna.lundberg.gu.se/cutter2/) allowed for the selection of four endonucleases (HindIII, SacI, BamHI and EcoRV) that were likely to generate distinct RFLP patterns among the different *bont*/B subtypes.

**Figure 1:**
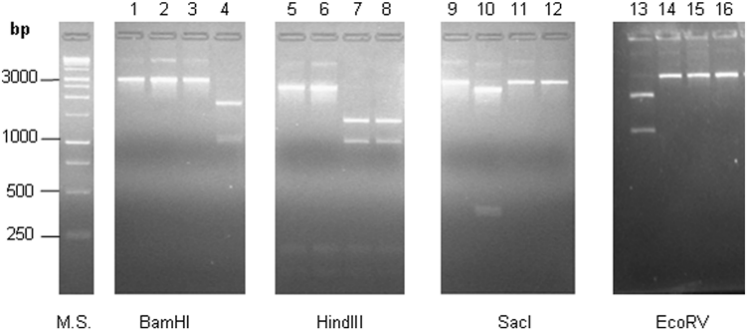
BamHI, HindIII, SacI and EcoRV restrictions of the *bont*/B PCR products obtained from strains CDC-1758 (lanes 1, 5, 9, 14); CDC-1828 (lanes 2, 6, 10, 15); CDC-1436 (lanes 3, 7, 11, 16); CDC-4848 (lanes 4, 8, 12); CDC-816 (lane 13). M.S. (Molecular standard, 1 kb Promega).

This approach was tested with 4 *C. botulinum* strains whose *bont*/B subtype was known and which were included in these experiments as references (Table 1): specifically, CDC-1758 for *bont*/B1 (GenBank Accession number: EF033127); CDC-1828 for *bont*/B2 (GenBank Accession number: EF051571); CDC-4848 (ATCC 25765) for non-proteolytic *bont*/B (GenBank Accession number: X71343); and CDC-1436 for bivalent *bont*/B (GenBank Accession number: AF295926) [4]. Once the PCR products from these strains were cut with the four restriction enzymes, each *bont*/B subtype was characterized by a specific restriction profile that was consistent with the one expected from the theoretical analysis (Figure 1). We lacked a reference for the *bont*/B3 subtype because the single *C. botulinum* strain that has been shown to harbor the *bont*/B3 subtype (strain CDC-795, GenBank Accession number: EF028400) [4] was not in our panel of strains. However, the theoretical restriction map analysis revealed an *Eco*RV restriction site at position 1828 of the 2807 bp region of the *bont*/B3 gene, which was absent from the analogous region of the other *bont*/B subtypes. It was therefore deduced that the PCR-RFLP strategy could be used for subtyping the *bont*/B gene.

**Table 1:** *C. botulinum* strains analyzed in this study, and genomic location of their *bont*/B and /A PCR-RFLP subtype genes.

Strain no^1^	Toxin type	State	Year	Source^2^	*bont*/B subtype^3^	*bont*/B genomic location	*bont*/A subtype	*bont*/A genomic location	plasmid size (kb) 4
CDC-555	B	OH	1976	corn	B1	plasmid			245
CDC-620	B	PA	1976	IB	B1	plasmid			245
CDC-628	B	CT	1976	uB	B1	plasmid			217
CDC-661	B	TN	1976	IB	B2	chromosome			
CDC-668	B	TN	1976	IB	B1	plasmid			245
CDC-706^5^	B	AK	1976	salmon	Bnp	plasmid			55
CDC-816	B	MI	1977	peppers	B3	chromosome			
CDC-1588	B	AZ	1977	IB	Bbv	plasmid			170
CDC-1632	B	PA	1977	IB	B1	chromosome			
CDC-1758	B	OR	1977	IB	B1	plasmid			245
CDC-1828	B	MO	1978	IB	B2	chromosome			
CDC-1852	B	CO	1978	FB	Bbv	plasmid			245
CDC-1872	B	MD	1978	IB	B1	plasmid			139
CDC-2064	B	PA	1978	IB	B1	plasmid			139
CDC-2094	B	MA	1978	AB	B1	plasmid			245
CDC-2113	B	NJ	1978	IB	B1	plasmid			245
CDC-2292	B	PA	1978	IB	B1	plasmid			245
CDC-2306	B	NJ	1978	IB	B1	plasmid			245
CDC-2312	B	PA	1978	IB	Bbv	n.d.^6^			
CDC-2329	B	DE	1978	IB	B1	plasmid			245
CDC-2358	B	MA	1978	uB	Bbv	plasmid			245
CDC-2586	B	KY	1979	IB	B2	chromosome			
CDC-2589	B	KY	1979	FB	B1	plasmid			139
CDC-2593	B	KY	1979	AB	B1	plasmid			245
CDC-2746	B	NY	1979	IB	B1	plasmid			245
CDC-2978	B	CO	1979	IB	Bbv	plasmid			170
CDC-4848^5^	B		ATCC	25765	Bnp	plasmid			55
CDC-5078	B	HI	1983	IB	Bbv	plasmid			245
CDC-5153	B	IN	1984	IB	B1	plasmid			139
CDC-5168	B	HI	1984	IB	Bbv	plasmid			245
CDC-5250	B	LA	1984	IB	B1	plasmid			245
CDC-5281	B	OK	1984	IB	B2	chromosome			
CDC-5323	B	DE	1985	IB	B1	plasmid			245
CDC-7699	B	LA	1990	FB	B1	plasmid			245
CDC-7827	B	NV	1991	IB	B1	n.d.^6^			245
MDb02	B	-	-	-	B1	plasmid			245
ISS-BC1	B	I	2000	olives	B2	chromosome			
ISS-BC2	B	I	2000	olives	B2	chromosome			
ISS-BC3	B	I	2001	truffles	B2	chromosome			
ISS-251	B	I	2003	chickpea	B2	chromosome			
ISS-257	B	I	2002	FB	B2	chromosome			
ISS-267	B	I	2003	IB	B2	chromosome			
ISS-274	B	I	2004	FB	B2	chromosome			
ISS-276	B	I	2002	honey	B2	chromosome			
ISS-306	B	I	2004	FB	B2	chromosome			
ISS-310	B	I	2004	FB	B2	n.d.^6^			
ISS-331	B	I	2004	IB	B2	chromosome			
ISS-333	B	I	2004	FB	B2	plasmid			245
ISS-338	B	I	2002	honey	B2	chromosome			
ISS-342	B	I	2004	tuna fish	B2	chromosome			
ISS-360	B	I	2005	peppers	B2	chromosome			
ISS-372	B	I	2005	beans	B2	chromosome			
ISS-378	B	I	2006	mushrooms	B2	chromosome			
ISS-388	B	I	2006	FB	B2	chromosome			
CDC-588	Ab	OH	1976	FB	Bbv	chromosome	A1	chromosome	
CDC-1436	Ab	UT	1977	IB	Bbv	plasmid	A2	plasmid	245
ISS-87	Ab	I	1995	FB	B3	plasmid	A2	plasmid	245
ISS-92	Ab	I	1993	FB	B1	plasmid	A2	plasmid	245
CDC-1634	A(B)	PA	1977	IB	Bbv	chromosome	A1	chromosome	
CDC-1727	A(B)	AK	1977	whale oil	Bbv	chromosome	A1	chromosome	
CDC-1807	A(B)	CO	1977	beans and franks	Bbv	chromosome	A1	chromosome	
CDC-4893	A(B)	IL	1983	FB	Bbv	chromosome	A1	chromosome	
CDC-5277	A(B)	WV	1984	IB	Bbv	chromosome	A1	chromosome	

1 CDC (Centers for Disease Control and Prevention, USA); ISS (Istituto Superiore di Sanità, Italy).

2 IB (infant botulism); FB (foodborne botulism); AB (animal botulism); uB (unknown botulism).

3 Bnp (non-proteolytic B); Bbv (bivalent B).

4 The size of the closest molecular standard bands are indicated.

5 Non-proteolytic *C. botulinum*.

6 n.d. = Not determined, because of DNA degradation.

All five *bont*/B PCR-RFLP subtypes were detected among the 63 *C. botulinum* strains that were tested (Table 2). *Bont*/B1 and /B2 were the most frequent PCR-RFLP subtypes and were identified in, respectively, 23 (36.5%) and 22 (34.9%) strains of the total 63 tested. *Bont*/B1 predominated among the US strains (22/43 strains), whereas *bont*/B2 was predominant among the Italian strains (18/20 strains). To date, the bivalent *bont*/B subtype has only been described in *C. botulinum* bivalent strains, which are designated as “bivalent” because they harbor two distinct *bont* genes [3], [4]. In this study the bivalent *bont*/B PCR-RFLP subtype was identified in 7 of the 9 bivalent *C. botulinum* strains tested: of these, 5 were *C. botulinum* type A(B) strains which only produce BoNT/A because their *bont*/B gene remains unexpressed [15], and 2 were *C. botulinum* type Ab strains which concomitantly produces both BoNT/A and /B, the latter in minor amounts [16]. Furthermore, the bivalent *bont*/B PCR-RFLP subtype was atypically detected in 7 *C. botulinum* type B strains that were non-bivalent, that is, negative for *bont* genes other than B when tested by PCR (data not shown). Hence, a total of 14 strains (22.2% of the 63 tested) displayed the bivalent *bont*/B PCR-RFLP subtype. Surprisingly, the remaining two bivalent *C. botulinum* type Ab strains (ISS-87 and ISS-92), both from Italy, displayed a *bont*/B1 and a *bont*/B3 PCR-RFLP subtype, respectively, rather than the expected bivalent *bont*/B subtype: since both strains had previously been shown to contain a *bont*/A2 gene [17], they represent two novel A2/B1 and A2/B3 bivalent variants. The *bont*/B3 PCR-RFLP subtype was detected in an additional *C. botulinum* type B strain, whereas the non-proteolytic *bont*/B PCR-RFLP subtype was only identified in the reference strain and one additional nonproteolytic *C. botulinum* type B strain.

**Table 2:** Distribution of *bont*/B PCR-RFLP subtype genes among 63 *C. botulinum* strains.

*bont*/B PCR-RFLP subtype	No. (%) of strains	No. of *C. botulinum* strains (total)					
		Type B (54)		Type Ab (4)		Type A(B) (5)	
		Italy (18)	USA (36)	Italy (2)	USA (2)	Italy (0)	USA (5)
B1	23 (36.5)		22	1			
B2	22 (34.9)	18	4				
B3	2 (3.2)		1	1			
bivalent B	14 (22.2)		7		2		5
nonproteolytic B	2 (3.2)		2				

### Genomic localization of the *bont*/B and /A subtype genes

Previous studies have demonstrated that Southern blot analysis of pulsed-field gels containing undigested genomic DNA from bacterial cells can be used to establish whether a certain gene is chromosomally or extra-chromosomally located [18], [19]. The same strategy, based on pulsed-field gel electrophoresis (PFGE) and subsequent hybridization with *bont*/A and /B specific gene probes, has also been applied to show plasmid carriage of the *bont*/A3, /A4, and bivalent *bont*/B subtype genes [10]. We used this PFGE Southern blot approach to define the genomic location of the *bont*/B genes for the total 63 *C. botulinum* strains included in this study (Table 1); the genomic location of the *bont*/A gene in the 4 type Ab and 5 type A(B) *C. botulinum* strains was analyzed as well.

Three of the 63 *C. botulinum* strains (CDC-7827, displaying the *bont*/B1 PCR-RFLP subtype; ISS-310, displaying the *bont*/B2 PCR-RFLP subtype; and CDC-2312, displaying the bivalent *bont*/B PCR-RFLP subtype) repeatedly produced DNA smears after PFGE, presumably due to enhanced DNAse activity: consequently, they were not suitable for the subsequent hybridization experiments, and the genomic location of their *bont* genes could not be determined by this technique.

The *bont*/B probe hybridized to the chromosomal band of 28 *C. botulinum* strains (47% of the total 60 PFGE-typeable strains tested). In the remaining 32 *C. botulinum* strains (53%), the *bont*/B probe hybridized to an extra-chromosomal band (Table 3). In particular, extra-chromosomal location was found for: 21/22 (95%) of the *bont*/B1 PCR-RFLP subtypes; 2/2 (100%) non-proteolytic *bont*/B subytpes; 7/13 (54%) bivalent *bont*/B subtypes, of which 6 atypically detected in non-bivalent *C. botulinum* strains (Table 1); 1/2 (50%) *bont*/B3 subtypes, the latter atypically detected in a bivalent *C. botulinum* type Ab strain (ISS-87); and 1/21 (5%) *bont*/B2 subtypes.

**Table 3:** Plasmid-borne *bont*/B PCR-RFLP subtype genes among *C. botulinum* strains.

*bont*/B PCR-RFLP subtype	No. of PFGE-typeable strains (n = 60)	Plasmid carriage (%) (n = 32)
B1	22	21 (95%)
B2	21	1 (5%)
B3	2	1 (50%)
bivalent B	13	7 (54%)
nonproteolytic B	2	2 (100%)

When the Southern blots of the PFGE gels containing the genomic DNA from the bivalent *C. botulinum* strains included in this study were stripped and rehybridized with a *bont*/A specific gene probe, a hybridization signal was observed at the same blot location that reacted with the *bont*/B specific gene probe, indicating that the *bont*/A and /B genes share the same genomic location in these strains. Specifically, both *bont*/A and /B probes hybridized to the chromosomal band of 6 bivalent *C. botulinum* strains, including all 5 *C. botulinum* type A(B) strains and 1 of the 4 *C. botulinum* type Ab strains (CDC-588): all 6 strains exhibited the bivalent *bont*/B PCR-RFLP subtype and had previously been shown to possess the *bont*/A1 subtype [17], [20]. In the remaining 3 bivalent *C. botulinum* type Ab strains (CDC-1436, ISS-87, and ISS-92), both *bont*/A and /B probes hybridized to the same extra-chromosomal blot location: the 3 strains exhibited *bont*/B1, /B3, and bivalent/B PCR-RFLP subtypes, respectively, and had previously been shown to possess the *bont*/A2 subtype [17], [20] (Table 1).

**Figure 2:**
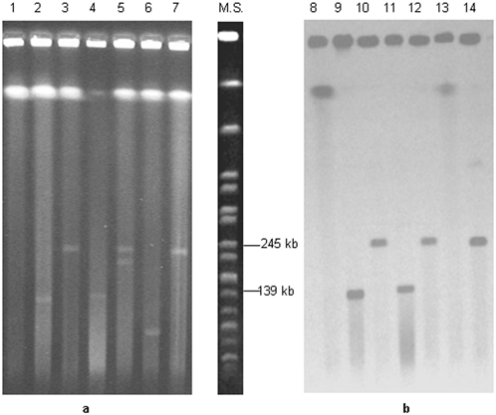
PFGE gel (a) and Southern blot membrane after hybridization with a *bont*/B gene probe (b). Strains: ISS-274 (lanes 1, 8); CDC-2589 (lanes 2, 9); CDC-2306 (lanes 3, 10); CDC-1872 (lanes 4, 11); CDC-5250 (lanes 5, 12); ISS-BC2 (lanes 6, 13); CDC-5323 (lanes 7, 14). M.S. (Molecular standard: DNA isolated from *Salmonella enterica* serotype Braenderup strain H9812 and restricted with *Xba*I) [23].

Figure 2 shows a PFGE gel stained with ethidium bromide and its Southern blot membrane after hybridization with a non-radioactive *bont*/B gene probe.

### Characterization and size determination of the extra-chromosomal bands

**Figure 3:**
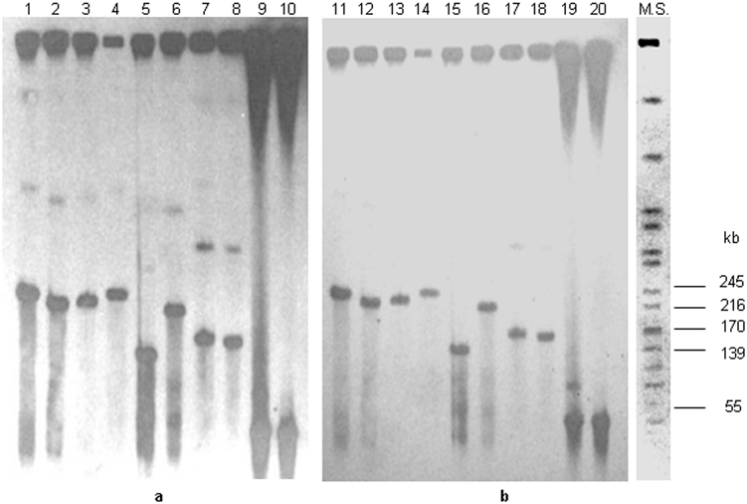
Southern blot membrane before (a) and after (b) S1 nuclease treatment. Strains: CDC-MDb2 (lanes 1, 11); CDC-1758 (lanes 2, 12); CDC-5078 (lanes 3, 13); CDC-1852 (lanes 4, 14); CDC-1872 (lanes 5, 15); CDC-628 (lanes 6, 16); CDC-1588 (lanes 7, 17); CDC-2978 (lanes 8, 18); CDC-706 (lanes 9, 19); CDC-4848 (lanes 10, 20). M.S. (Molecular standard: DNA isolated from *Salmonella enterica* serotype Braenderup strain H9812 and restricted with *Xba*I) [23].

The PFGE Southern blot assay can also be used to estimate the size of extra-chromosomal hybridization bands, when present; however, large circular DNA molecules can exhibit anomalous migration during PFGE [21], [22]. As shown in Figure 3a, the DNA preparations from several *C. botulinum* isolates displayed double bands after hybridization with the *bont*/B gene probe: this could indicate either that these isolates carry multiple copies of the *bont*/B gene on two distinct extra-chromosomal elements of different sizes or that the same extra-chromosomal element harboring the *bont*/B gene exists as variable forms whose mobility differs under PFGE conditions. The latter hypothesis is consistent with the diverse mobility of the isoforms of large (>100 kb) circular plasmids: *i*) open circular forms, which remain trapped in the sample wells of pulsed-field gels; *ii*) closed supercoiled forms, which move slowly under PFGE conditions; and *iii*) linear forms, which migrate at rates that allow the size of the plasmids to be accurately determined [22]. Based on these considerations, the double extra-chromosomal bands that we observed in some DNA samples might correspond to the linear and supercoiled forms of the same plasmids. To test this hypothesis, the PFGE plugs were treated with S1 nuclease, which enzymatically converts all plasmid forms into linear forms [22]. The S1 nuclease treatment caused the disappearance of the slower of the two bands present in the DNA samples, indicating that this band was probably the supercoiled plasmid; the mobility of the faster band was not affected by S1 nuclease treatment, which is consistent with the behavior expected for the linear plasmid [22]. Whether or not linear and supercoiled plasmid forms are present depends on the age of the culture [22], which is consistent with the appearance of the double hybridization bands in some, but not all, of our DNA samples: in fact, the DNA preparations that we used were obtained from bacteria collected from overnight cultures, whose growth phases were not uniform (see the Materials and Methods section). Other DNA samples displayed single extra-chromosomal bands after hybridization with the *bont*/B gene, which presumably corresponded to the linear plasmid forms; indeed, their mobility did not change after S1 nuclease treatment.

As illustrated in Figure 3b, PFGE resolved at least five differently sized *bont*/B-carrying plasmids among the 32 *C. botulinum* isolates. They ranged from ∼55 kb to ∼245 kb, as determined by comparison with a molecular standard (DNA isolated from *Salmonella enterica* serotype Braenderup strain H9812 and restricted with *Xba*I) [23]. Specifically, a plasmid greater than the 245 kb band of the molecular size marker was detected in 23 *C. botulinum* strains (72% of the 32 strains harboring a plasmid-borne *bont*/B gene). Of these, 16 displayed the *bont*/B1 PCR-RFLP subtype, 5 displayed the bivalent *bont*/B PCR-RFLP subtype, and 2 displayed the *bont*/B2 and *bont*/B3 PCR-RFLP subtypes (Table 4). A plasmid in line with the 139 kb band of the molecular standard was observed in 4/32 (12.5%) *C. botulinum* strains, all of which exhibited the *bont*/B1 PCR-RFLP subtype. Furthermore, plasmids close in size to the 170 kb and 55 kb bands of the molecular standard were observed in 2 *C. botulinum* strains each; both ∼170 kb plasmids carried the bivalent *bont*/B PCR-RFLP subtype, whereas both ∼55 kb plasmids carried the non-proteolytic *bont*/B PCR-RFLP subtype. Finally, a unique plasmid of approximately 217 kb was detected in a single strain displaying the *bont*/B1 PCR-RFLP subtype. The plasmids harboring both *bont*/A and /B genes that were detected in 3 bivalent *C. botulinum* strains were >245 kb.

**Table 4:** Molecular size of the plasmids carrying the different *bont*/B subtype genes.

Plasmid Size (kb)^a^	*bont*/B1	*bont*/B2	*bont*/B3	bivalent *bont*/B	Nonproteolytic *bont*/B
∼245	16	1	1	5	−
∼217	1	−	−	−	−
∼170	−	−	−	2	−
∼139	4	−	−	−	−
∼55	−	−	−	−	2

a The plasmid sizes were deduced by comparison with a molecular standard [23].

Slight differences between the sizes of plasmids included in the same group were observed.

## Discussion

Subtype diversity within BoNT serotypes can significantly impact their receptor binding, target affinity and antibody recognition; the latter issue may be critical for the development of effective neutralizing antibodies [3]. However, little is known about the distribution of the BoNT subtypes and their encoding genes among *C. botulinum* strains with different origins. We previously analyzed the *bont*/A gene of a set of *C. botulinum* strains using a PCR-RFLP approach [17]: although this method can only detect a few single nucleotide polymorphisms that are recognized by specific restriction enzymes, it correctly identified the *bont*/A subtype genes, as subsequently shown by the complete nucleotide sequencing of some of those genes [24], [25]. In the current study, we adopted a similar PCR-RFLP approach for subtyping the *bont*/B gene in a panel of *C. botulinum* strains from different origins (clinical forms of botulism and foods), countries, and periods. Our results revealed that the *bont*/B2 PCR-RFLP subtype was more prevalent among *C. botulinum* strains from Italy, whereas the *bont*/B1 PCR-RFLP subtype prevailed among strains from the US, confirming earlier suggestions by Hill *et al.* [4]. A similar geographic distribution has been found for *bont*/A subtypes, with *bont*/A2 more prevalent in Italy and *bont*/A1 more prevalent in the US [17]. These results could reflect a variation in the evolutionary history of both *bont*/A and /B subtypes. Furthermore, no correlation was observed between any specific *bont*/B subtype and the clinical or food source of the strains or the period in which the strains were isolated, as previously shown for *bont*/A subtypes [17].

Unexpectedly, we found that the bivalent *bont*/B PCR-RFLP subtype was not restricted to the bivalent *C. botulinum* strains and was detected in several non-bivalent type B strains; we also found that two bivalent *C. botulinum* type Ab strains exhibited the *bont*/B1 and /B3 PCR-RFLP subtypes. These results could indicate that the *bont*/B genes of these peculiar *C. botulinum* strains could be previously unrecognized subtype genes, although this would need to be confirmed with sequencing. Alternatively, the finding that some *C. botulinum* strains display *bont*/B subtypes other than the expected ones could be indicative of mobilization of the *bont*/B gene among strains at some point during evolution. Circumstantial evidence that the *bont*/B gene is mobile and might have been transferred among progenitor strains already exists, in particular: i) BoNT/B can be formed by *C. botulinum* strains of different clostridia groups [2]; ii) BoNT/B is produced along with another BoNT type by some *C. botulinum* strains, such as those of type Ab, Ba, and Bf [16]; iii) a silent *bont*/B gene is present in the genome of *C. botulinum* type A(B) strains [15]; and iv) toxigenic *C. botulinum* type B and its non-toxigenic derivatives have been isolated from the same samples [26]. Although there is no direct evidence of the mobilization of the *bont*/B gene, the recent demonstrations that the gene is plasmid-encoded in certain *C. botulinum* strains further support this hypothesis: indeed, plasmids may play an important role in mediating genetic transfer within and among bacterial genomes [27].

With regard to the genomic location of the *bont*/B1, /B3 and bivalent /B subtype genes atypically detected in the *C. botulinum* strains mentioned above, our results showed that the *bont*/B genes were extra-chromosomally located in all of these strains, though for one strain the bivalent *bont*/B subtype gene could not be localized because of consistent DNA degradation. Analysis of the genomic location of the *bont*/B genes was then extended to all *C. botulinum* strains included in this study: surprisingly, extra-chromosomal elements carrying the *bont*/B gene were detected in most (53%), with no apparent relationship with their origin.

We assumed that the *bont*/B-carrying extra-chromosomal elements were circular plasmids, based on the agreement between their PFGE migration and the mobility behavior predicted for the supercoiled and linear forms of circular plasmids [22]. Furthermore, some of the extra-chromosomal elements were similar in size to those of the *bont*/B-encoding plasmids determined to date [i.e., 270 kb for pCLJ of strain 657, 149 kb for pCLD of strain Okra [12], and 48 kb for pCLL of strain Eklund 17B (GenBank Accession number CP001057)], though two previously unreported sizes were also revealed in this study. However, we predicted the plasmid sizes by comparison with a molecular standard, whereas an accurate size determination would require the complete nucleotide sequencing of the plasmids.

Our results indicate that all *bont*/B subtypes can be located on plasmids and that a *C. botulinum* strain can only carry *bont*/B plasmids of a single size. Specifically, of the *bont*/B plasmids identified for 32 *C. botulinum* strains, 24 were greater than 200 kb, and the remaining 8 ranged from approximately ∼55 kb to ∼170 kb: assuming that the *C. botulinum* chromosome is about 3.9 Mb [9], such plasmids would constitute a variable proportion (from 0.1% to 6%) of the genomes of the strains harboring them. The largest plasmids (∼245 kb) were associated with all *bont*/B subtypes, except for the non-proteolytic *bont*/B subtypes, which were only associated with the smallest plasmids (∼55 kb). Notably, one of the non-proteolytic *bont*/B subtypes belonged to strain CDC-4848 (or ATCC 25765), which was found to correspond to strain Eklund 17B: this strain has plasmid sequence of ∼48 kb (GenBank Accession number CP001057), which is similar to the ∼55 kb in our study. The intermediate-sized plasmids detected in the present study were associated with either the *bont*/B1 or the bivalent *bont*/B subtypes, suggesting that both subtypes can reside on plasmids of different sizes. For the strains whose *bont*/B gene was plasmid-borne, no chromosomal band hybridized with the *bont*/B gene probe, indicating that the plasmids did not integrate with the chromosome.

Remarkably, 95% of the *bont*/B1 subtypes resided on plasmids, whereas the same percentage of the *bont*/B2 subtypes resided on the chromosome. Although the biological significance of this finding is unclear, it supports the hypothesis of diverse evolutionary pathways for the *bont*/B1 and /B2 subtypes, as hypothesized above based on their geographic separation.

For three of the nine bivalent *C. botulinum* strains (all three of type Ab), the *bont*/A and /B genes were located on the same plasmid, whose size was similar to that of plasmid pCLJ (270 kb) of the bivalent *C. botulinum* type Ba strain 657 [10], [12]. However, for the other 6 bivalent *C. botulinum* strains [one of type Ab and 5 of type A(B)], the *bont*/A and /B genes were located in the chromosome. The finding that the *bont*/A and /B genes share the same genomic location (whether it was the chromosome or the plasmid) suggests that they are structurally linked. The specific location could be related to the *bont*/A subtype: in fact, all three of the strains showing plasmid location of the genes had *bont*/A2 and all 6 of the bivalent strains with chromosome location had *bont*/A1 [17], [20]. However, this hypothesis would need to be tested with more bivalent strains.

To determine the reasons for which the genomic location of the *bont*/A and /B genes varies among the different *C. botulinum* strains, the *bont* plasmids identified in the present study will need to be characterized at the sequence level. Partial mobile-enabling sequences, such as insertion sequences (IS), have been detected downstream of the *bont*/B and /A genes in plasmids pCLJ, pCLD and pCLK; when chromosomal, the *bont* genes are flanked by both upstream and downstream IS-like elements, which might be indicative of transposon integration [12]. The association between remnants of IS elements and the *bont*/A and /B genes suggests that these genes could have been mobilized and stably inserted into the chromosome or plasmid, depending on the recipient *Clostridium* ancestor strain [28]. The same mechanism might have contributed to the dissemination of the *bont* genes among heterogeneous groups of clostridia; in this respect, the ability of the BoNT-encoding plasmids to undergo conjugation or other types of genetic transfer should also be investigated.

Notably, many similarities have previously been reported among the BoNT/B of *C. botulinum*, the BoNT/G of *C. argentinense*, and the tetanus neurotoxin (TeNT) of *C. tetani*, which is also plasmid-encoded [29]: in particular, these neurotoxins have a high nucleotide and amino-acid sequence homology [4], [30], and they cleave the same presynaptic membrane protein synaptobrevin, though at different peptide bonds [1]. Furthermore, BoNT/B and /G recognize the same neuronal receptors [31], [32]. The finding that the *bont*/B gene can be plasmid-borne, like the *bont*/G and *tent* genes, strongly supports the hypothesis that they descend from a common ancestor.

A future challenge will be to determine whether the BoNT-encoding plasmids play a role in the development and/or flexibility of *C. botulinum*, thus potentially increasing its adaptability to certain niches, such as specific environments and food matrixes, and ultimately contributing to the disease of botulism.

## Materials and Methods

### Clostridia strains and culture conditions

A total of 63 *C. botulinum* strains were used in this study (Table 1). Of these, 20 were from the culture collection of the National Reference Center for Botulism, Istituto Superiore di Sanità (ISS), Rome, Italy, and had been isolated between 2000 and 2006; the remaining 43 strains, isolated in the United States from 1976 to 1990, were generously provided by Charles Hatheway of the Botulism Laboratory, Centers for Disease Control and Prevention (CDC), Atlanta, GA.

Forty-six strains were of clinical origin, specifically, 45 were from distinct cases of human botulism and 1 from animal botulism; 1 strain (ATCC 25765) was from a marine sediment of the West Coast of the US; 1 strain was of unknown origin; and the remaining 14 strains were from different types of foods.

Fifty-four strains were *C. botulinum* type B (i.e., they produced BoNT/B and were positive for the *bont*/B gene when tested by PCR) [15]; 9 other strains had been shown to contain, in addition to the *bont/A* gene, a *bont/B* gene in their genome, which was either silent [*C. botulinum* A(B)] (CDC-1634, -4893, -5277, -1807, and -1727) or expressed [*C. botulinum* Ab] (CDC-588, -1436, ISS-87, and -92) [15], [17]. Clostridia stock cultures were checked for purity on egg yolk agar (EYA) plates (Oxoid, Basingstoke, England). Single colonies were transferred from the plates to 10 ml of TPGY broth (5% Trypticase, 0.5% peptone, 0.4% glucose, 2% yeast exctract, 1% L-cysteine hydrocloride monohydrate) and grown overnight at 37°C, under anaerobiosis (GasPack jars, Oxoid).

For the PCR experiments, 1 ml from each overnight broth-culture was centrifuged and washed with 1XTE buffer (10 mM Tris, pH 7.4, 1 mM EDTA); the pellets were re-suspended in 1 ml of sterile distilled water, and 5 µl of the cell suspensions was used as DNA template in each PCR reaction mixture. Nine milliliters of the overnight TPGY broth-cultures was used for the PFGE experiments.

### PCR restriction fragment length polymorphism (RFLP) subtyping of the *bont*/B gene

Primers BS1 (5′ AGTTTGCATATCAGATCCTAA 3′) and BS2 (5′ AACGATGAATACCAATCAATCC 3′) were selected in conserved regions of the aligned *bont*/B gene sequences available from the GenBank database, to generate a PCR product encompassing nucleotides 920 to 3727. PCR products were amplified in a programmable thermal cycler (M.J. Research, model PTC100, Waltham, MA) using the Expand High Fidelity PCR System (Roche Diagnostics, Penzberg, Germany) according to the manufacturer's instructions with the following amplification parameters: 1 cycle of 2 min at 94°C; 30 cycles consisting of 15 sec at 94°C, 30 sec at 50°C, 4 min at 68°C with 5 sec of increment/cycle; and a final cycle at 68°C for 7 min. The PCR products were resolved by TAE gel electrophoresis, purified by a DNA gel extraction kit (Montage, Millipore Corporation, Bedford, MA, USA), and digested separately with 10 units of BamHI, HindIII, SacI and EcoRV (New England Biolabs, Ipswich, MA). All restrictions were performed at least twice.

### Pulsed-field gel electrophoresis (PFGE) and S1 nuclease treatment of plugs

Agarose plugs containing the genomic DNA from the bacterial cultures were prepared as previously described [17]. The DNA isolated from the *Salmonella enterica* serovar Braenderup strain H9812 and restricted with *Xba*I (Roche Diagnostics) served as the molecular standard [23].

For the treatment with S1 nuclease, single slices of some agarose plugs were washed twice in S1 nuclease buffer (50 mM NaCl, 30 mM sodium acetate [pH 4.5], 5 mM ZnSO_4_) and incubated with 1 unit of *Aspergillus oryzae* S1 nuclease (MBI Fermentas, Vilnius, Lithuania) in 200 µl of S1 buffer for 10 min at 37°C. PFGE of undigested or S1-digested DNA was carried out in a contour-clamped homogeneous electric field apparatus (CHEF Mapper apparatus, BioRad Laboratories, Hercules, CA). A constant temperature of 14°C was used, and the electrophoresis parameters were as follows: voltage of 6 V/cm, an angle of 120, and switch times of 4 to 40 sec (linear ramping factor), for 22 h. Gels were stained with ethidium bromide and visualized in a GelDoc 2000 apparatus (Bio-Rad Laboratories).

### Probes preparation and Southern hybridization

A 592 bp fragment of the *bont*/B gene and a 268 bp fragment of the *bont*/A gene were PCR labeled with digoxigenin (DIG), using primers described elsewhere [10] and a non-radioactive DNA probe labeling kit (PCR DIG Probe Synthesis Kit, Roche Diagnostics GmbH, Mannheim, Germany). The DNA was transferred from PFGE gels to positively charged nylon membranes (Hybond-N+, Roche) by overnight capillary transfer with buffer 20× SSC (3.0 M sodium chloride, 0.3 M sodium citrate, pH 7.0). After transfer, nylon membranes were hybridized with either *bont*/B or *bont*/A gene probes at 42°C for 18 h in a DIG Hyb Solution (Roche).

A chemiluminescence-based method was used to detect probe-target hybrids, according to the manufacturer's instructions (Roche). Briefly, the membranes were blocked for 30 min; 20 µl of anti-digoxigenin-AP Fab-fragments (15 U/20 ml) (Roche) were added to the blocking solution; and the membranes were incubated for 30 min at room temperature. After equilibration in detection buffer, the membranes were incubated with chemiluminescent substrate CSPD in a Hypercassette (Amersham Pharmacia Biotech, Milan, Italy) and exposed to CL-XPosure film (Pierce Chemical, Rockford, IL).

For membrane stripping and rehybridization, a previously hybridized membrane was rinsed with distilled water and then soaked three times for 30 min in 0.2 M NaOH containing 0.1% sodium dodecyl sulfate at 37°C, to remove the bound probe. The membrane was washed for 15 min in 2× SSC and then hybridized with a second probe.
